# Facial emotion processing in patients with borderline personality disorder as compared with healthy controls: an fMRI and ECG study

**DOI:** 10.1186/s40479-024-00245-4

**Published:** 2024-02-16

**Authors:** Monika Radimecká, Adéla Látalová, Martin Lamoš, Martin Jáni, Patrik Bartys, Alena Damborská, Pavel Theiner, Pavla Linhartová

**Affiliations:** 1 Department of Psychiatry, University Hospital Brno and Faculty of Medicine, Masaryk University, Jihlavská 340/20, Brno, 625 00 Czech Republic; 2 Brain and Mind Research Program, Central European Institute of Technology, Masaryk University, Brno, Czech Republic

**Keywords:** Borderline personality disorder, Facial emotion processing, Negative facial expressions, Faces task, fMRI, Heart rate variability

## Abstract

**Background:**

Maladaptive behaviors and interpersonal difficulties in patients with borderline personality disorder (BPD) seem connected to biased facial emotion processing. This bias is often accompanied by heightened amygdala activity in patients with BPD as compared to healthy controls. However, functional magnetic resonance imaging (fMRI) studies exploring differences between patients and healthy controls in facial emotion processing have produced divergent results. The current study explored fMRI and heart rate variability (HRV) correlates of negative facial emotion processing in patients with BPD and healthy controls.

**Methods:**

The study included 30 patients with BPD (29 females; age: M = 24.22, SD = 5.22) and 30 healthy controls (29 females; M = 24.66, SD = 5.28). All participants underwent the “faces” task, an emotional face perception task, in an fMRI session simultaneously with ECG. In this task, participants are presented with emotional expressions of disgust, sadness, and fear (as a negative condition) and with the same pictures in a scrambled version (as a neutral condition).

**Results:**

We found no differences in brain activity between patients with BPD and healthy controls when processing negative facial expressions as compared to neutral condition. We observed activation in large-scale brain areas in both groups when presented with negative facial expressions as compared to neutral condition. Patients with BPD displayed lower HRV than healthy controls in both conditions. However, there were no significant associations between HRV and amygdala activity and BPD symptoms.

**Conclusion:**

The results of this study indicate no abnormal brain activity during emotional facial processing in patients with BPD. This result contrasts with previous studies and more studies are needed to clarify the relationship between facial emotion processing and brain activity in patients with BPD. Possible reasons for the absence of brain activity differences are discussed in the study. Consistent with previous findings, patients showed lower HRV than healthy controls. However, HRV was not associated with amygdala activity and BPD symptoms.

## Background

Emotional dysregulation, usually described as an inability to control and regulate one’s affective state, is one of the core features in borderline personality disorder (BPD) [1]. It is linked with difficulties in the personal experience of emotion and with the perception of emotion in others [2, 3]. Given that facial expressions are fundamental emotional stimuli for everyday social functioning [4], biased processing of emotional faces in patients with BPD may lead to frequently observed maladaptive behaviors and interpersonal problems, including loneliness, conflictual relationships, and fear of abandonment.

Mitchell et al. [5] reviewed 25 studies (*N* = 1279) exploring differences in facial emotion processing between patients with BPD and healthy controls (HC). They found conclusive evidence of a tendency in patients to interpret ambiguous and neutral expressions as negative expressions. However, the findings for negative and positive expressions have been divergent. Whereas some studies found impaired accuracy in recognizing a wide range of negative expressions [6], others found impairment only for specific expressions such as fear or disgust [7–9] or no impairment at all [10, 11]. Furthermore, no recognition difficulties were found in full-intensity happy expressions [12]. Patients were more accurate in recognizing happy faces than angry, sad, and neutral faces and displayed even greater accuracy than HC [6, 13].

Processing emotional expressions requires diverse psychological effort driven by multiple neural structures [14]. Fusar-Poli et al. [15] conducted a meta-analysis on 105 functional magnetic resonance imaging (fMRI) studies exploring the neural correlates of human facial expression processing in healthy respondents (*N* = 1600). They found increased activation of various visual areas (fusiform gyrus, inferior and middle occipital gyri), limbic areas (amygdala and parahippocampal gyrus, posterior cingulate gyrus), temporal areas (middle and superior temporal gyrus), temporoparietal areas (parietal lobule, middle temporal gyrus, insula), prefrontal areas (medial frontal gyrus), and in the putamen and cerebellum. Although the specific interactions between these areas have not been clarified, it is hypothesized that the initial perceptual processing of faces occurs in the occipital and temporal lobes, which construct detailed representations from facial features. Subsequently, a set of brain areas, including the amygdala and orbitofrontal cortex, links the perceptual representation of the face to the meaning of the signaled emotion [15]. The amygdala is a crucial brain area associated with BPD symptoms, and studies often report its hyperactivity during emotional processing in patients [16]. Its activity may also be altered during the processing of facial expressions.

Recently, there has been an increase in fMRI studies exploring brain activity differences in patients with BPD. The review by Mitchell et al. [5] also described the differences in neural activation during facial processing between HC and patients with BPD. Heightened activity of the left and right amygdala in patients during the presentation of facial stimuli was found across studies. Specifically, patients with BPD exhibited increased amygdala activity in response to neutral stimuli and heightened amygdala activity in response to fear. A later study by Wrege et al. [17] presented images of faces with neutral and moderately fearful expressions (50%) or intensively (100%) fearful expressions to 39 patients with BPD and 25 HC. Besides higher amygdala activation, the results revealed higher activations when viewing neutral faces (neutral faces > fixation cross) in the right temporal pole, hippocampus, pallidum, and orbitofrontal cortex in the patients with BPD as compared with the HC. There were no group differences in whole-brain activation when viewing fearful facial expressions of both intensities (moderately fearful faces > fixation cross; intensively fearful faces > fixation cross). However, the region of interest (ROI) analysis (intensively fearful > neutral; moderately fearful > neutral faces) focused on the amygdala and hippocampus revealed higher bilateral amygdala and right hippocampus activity in response to moderately fearful expressions in patients with BPD as compared with HC. Lamers et al. [18] showed patients with BPD (*N* = 20) and HC respondents only fearful faces stimuli. During this task, the patients with BPD showed hyperactivation of the frontostriatal, posterior cingulate cortex, and posterior parietal brain areas compared to HC (negative > neutral faces). ROI analysis also suggested higher activation in the emotion regulation network (amygdala, hippocampus, anterior cingulate cortex; ACC, insula, and dorsolateral prefrontal cortex) in the patients with BPD (negative > neutral faces) when compared to HC.

As for other negative emotions, Donegan et al. [19] presented patients with BPD (*N* = 15) and HC with sad facial expressions. They found heightened left amygdala activity in response to sadness (sad faces > fixation point) in patients with BPD as compared with HC. Another study by Minzenberg et al. [20] used angry expressions in a sample of patients with BPD (*N* = 12) and HC. In comparison to HC, the patients exhibited less activity in the bilateral amygdala, but more activity in the bilateral rostral ACC in response to angry expressions (anger > neutral faces). Baskin-Sommers et al. [21] also presented respondents (N_BPD_ = 13) with angry expressions. In this study, patients with BPD demonstrated higher activity in response to anger in the left superior frontal gyrus (angry > neutral faces) relative to HC.

No unusual amygdala activity or typical patterns in brain activation were found regarding the processing of happy expressions in fMRI studies. Baskin-Sommers et al. [21] (N_BPD_ = 13) found increased activity of the left inferior and decreased activity of the left superior frontal brain areas (happy > neutral faces); a more recent study by Lamers et al. [22] (N_BPD_ = 22) did not replicate these findings. Besides the activity of temporal, limbic, and occipital areas in both groups, hyperactivation of the bilateral caudate was observed in the patients with BPD (happy > neutral faces).

With respect to the heterogeneous results of fMRI studies in this area, other methods might be useful to identify differences in facial processing between patients with BPD and healthy people. There has been increasing interest in heart rate variability (HRV) as a physiological marker of emotional stability and flexibility. HRV is the inconsistency of time intervals between individual heartbeats resulting from the balance between the sympathetic and parasympathetic branches of the autonomic nervous system [23]. Alteration of HRV emerges from the loss of balance between these branches and the constant need of the heart to adapt to changing circumstances. High HRV is associated with better adaptation to environmental stressors and emotional stimuli, whereas low HRV is associated with emotional dysregulation and worse ability to control mental and behavioral impulses [23, 24].

Fluctuations of HRV (as an index of cerebral autonomic regulation) are associated with functional changes between and within brain regions included in the central autonomic network (CAN; prefrontal cortex, anterior cingulate cortex, insula, amygdala, periaqueductal gray, pons, and medulla) [25–27]. Although imaging studies have pointed out that changes in the neural activity of the amygdala, claustrum, thalamus, and hypothalamus contribute to HRV [25, 28, 29], less is known about their relationship during emotion regulation and specifically facial emotion processing. Steinfurth et al. [30] explored the relationship between habitual resting state vmHRV and neural activity during explicit emotion regulation in healthy controls (*N* = 24). The results showed an association between vmHRV and the activity of the prefrontal cortex (PFC) and amygdala during emotion regulation of unpleasant emotions. The group with high vmHRV displayed modulated activation of the right amygdala during the reappraisal strategy, and the group with low vmHRV displayed modulated activation of the right amygdala only when using the response modulation strategy. Similarly, respondents with high vmHRV displayed increased activation of the right PFC when regulating unpleasant emotion with reappraisal, whereas low vmHRV respondents displayed the same activation of PFC when using response modulation. Based on these results, the author suggested that different levels of vmHRV are associated with different patterns of brain activity during emotion regulation. A later study by Miller et al. [31] assessed neural activity and resting HRV while observing and imitating emotional faces in healthy respondents (*N* = 41). They found negative correlation between resting HRV and activation in the mirror neuron system, insula, and amygdala during observation, but not the imitation of emotional faces. Thus, the results supported the idea, that resting HRV is linked to neural sensitivity to other’s emotional cues, such as facial expressions.

Only a few studies have explored the alteration of HRV in patients with BPD. Koenig et al. [32] performed a meta-analysis on five studies (*N* = 200) focused on vagally-mediated HRV (vmHRV) in patients with BPD and HC. Results revealed lower vmHRV in patients with BPD relative to HC in resting state. Later studies found a significant relation between reduced HRV and greater BPD symptom severity together with worsened psychosocial functioning and higher emotional dysregulation, negative affective state, and depressive symptomatology [33–35]. Therefore, differences associated with emotional dysregulation between patients with BPD and HC may be apparent in HRV.

To our knowledge, there has not yet been a study reporting HRV in patients with BPD during facial emotion processing. However, Maiß et al. [36] explored HRV during the approach-avoidance task (AAT) in 42 patients with BPD. In this task, respondents are presented with happy and angry expressions and must pull or push these pictures according to their reaction. The results indicated a relationship between low HRV and an attenuated approach to angry faces with an averted gaze, suggesting that patients have a tendency to avoid this facial expression.

To summarize, patients with BPD are consistently reported to show deficits in processing neutral or ambiguous facial expressions, which they tend to interpret as negative. This bias is usually accompanied by higher amygdala activity in fMRI studies. However, the findings of fMRI studies regarding facial processing of other emotional valence have been inconsistent. Some studies found heightened left amygdala activity in patients with BPD in response to sad expressions [19]; others found lowered bilateral amygdala activity and heightened activity of the bilateral rostral ACC or left superior frontal gyrus in response to angry expressions [11, 21]. In this study, we explored fMRI and HRV correlates of processing facial expressions with negative valence since patients often experience difficulties in negative social situations. For this purpose, we used the “faces” task [37]. All respondents were presented with negative facial expressions (disgust, sadness, and fear) and with scrambled pictures as a control condition during fMRI with simultaneous electrocardiography (ECG) measurement. We did not use neutral expressions as a control condition because there is evidence of altered neural activation while viewing neutral faces in patients with BPD as compared to HC; this could diminish the activation differences associated with negative facial stimuli [17]. We used self-report questionnaires to assess fundamental symptoms of BPD (emotional dysregulation, rejection sensitivity, dissociation, and childhood trauma) that might affect emotional experiences during face processing in patients with BPD. We hypothesized that there are differences in neural activity between patients with BPD and HC while processing negative faces as compared to during a control condition. Based on previous studies, we specifically expected higher amygdala activity in the patients with BPD. We presumed that patients with BPD exhibit lower HRV than HC while viewing negative faces based on the previously found relationship of low HRV with emotional dysregulation. Lastly, in accordance with our previous suggestions, we expected higher amygdala activity to be associated with lower HRV during the processing of facial expressions in BPD patients.

## Methods

### Participants

This study was part of more extensive research exploring the neural mechanisms of dialectical behavioral therapy (DBT) in patients with BPD. The research was conducted in accordance with the Declaration of Helsinki and was approved by the Ethics Boards of the Faculty of Medicine, Masaryk University, and University Hospital Brno.

The current study was conducted before DBT treatment. We included 30 patients with BPD (29 females) and 30 matched HC (29 females). Patients with BPD were recruited from outpatient treatment at the Department of Psychiatry of the University Hospital Brno. Inclusion criteria were: 1) at least five out of the nine DSM-V criteria for BPD and 2) one or more suicide attempts and/or non-suicidal self-injury episodes in the past 6 months. Exclusion criteria were psychotic disorder, severe neurological disorder, or any contraindication to fMRI scanning. HC were recruited via social media advertisements. They were matched with patients in age (same age +/− 2 years), gender, and education (same level +/− one level). Educational levels were primary school, lower secondary, higher secondary, and university. Healthy controls had no current or lifetime psychiatric diagnosis.

Most patients (90%) also met the criteria for other psychiatric disorders, such as major depressive disorder (MDD) (50%), social anxiety disorder (33.3%), posttraumatic stress disorder (PTSD) (23.3%), substance use disorder (alcohol = 36.7%; drugs = 16.7%), obsessive-compulsive disorder (OCD) (13.3%), and panic disorder (13.3%). We also included medicated patients (83.3%). All participants were instructed not to take any sedative medication 24 hours before the fMRI session. They were allowed to take their other prescribed medication, consisting mainly of antidepressants and antipsychotics.

Since medication may alter neural activity [37, 38] we made a medication index to assess the possible effects of patient medication on neural activity. The medication index was made as an ordinary scale of medication according to Bartečků et al. [39]: 0 = no medication, 1 = one or more drugs at lower than the therapeutic dose, 2 = one drug at the therapeutic dose, 3 = more drugs with one at the therapeutic dose, 4 = more drugs at the therapeutic dose. Since major depressive disorder is one of the most common conditions that occur alongside BPD [40] and most prescribed medication in BPD patients includes antidepressants and antipsychotics [41, 42], we chose therapeutic doses of these two types of medication recommended for major depressive disorder. Therapeutic doses of antidepressants were specified according to the Antidepressant Treatment History Questionnaire (ATRQ; [43]). Therapeutic doses of antipsychotics were determined according to Wang et al. [44]. The medication index was then used as a covariate in statistical analyses.

### Procedure

All of the participants were informed about the study before the assessments and gave their written informed consent. On the first day, participants attended a semi-structured interview with a trained psychiatrist and completed self-report questionnaires on BPD symptoms. The next day, they performed three tasks during fMRI, starting with the “faces” task. Then participants underwent the Neurofeedback task (assessing the emotion regulation ability) and the Cyberball task (assessing rejection sensitivity). The whole assessment took about two and a half hours. This study presents the fMRI data from the “faces” task and its relation to the self-report questionnaires. Results from the Cyberball task in the same sample were published in Látalová et al. [45] study.

### Measurements

The BPD diagnosis was assessed by a trained clinician using the Structured Clinical Interview for DSM-V Personality Disorders [46].

Self-report questionnaires were used to assess the severity of BPD symptoms (Borderline Symptom List; BSL-23 [47]; Czech version: [48], emotional dysregulation (Difficulties in Emotion Regulation Scale Short Form; DERS-SF [49]; Czech version: [50], rejection sensitivity (Rejection Sensitivity RS-Adult questionnaire; A-RSQ [51], and dissociation (Multiscale Dissociation Inventory; MDI [52]. We also assessed childhood trauma (Childhood Trauma Questionnaire CTQ [53]; Czech version: [54]). Since there is no validated Czech version of the A-RSQ and MDI questionnaires, we translated these questionnaires using the back-translation method.

### Experimental task

We used the exact version of the “faces” task according to Paret et al. [37]. Participants viewed pictures of faces (12 actors and 12 actresses) with emotional expressions of disgust, sadness, and fear (negative condition; NgC) from the Warsaw Set of Emotional Facial Expression Pictures (http://www.emotional-face.org/), and the same pictures in a scrambled version (neutral condition; NC). In all, 72 emotional faces and 72 scrambled faces were used. The pictures were presented in 24 blocks (12 NgC and 12 NC), with the order pseudorandomized with maximally two identical conditions following each other. Every block consisted of a sequence of six faces, each presented for 3 seconds. Within each block, faces with different emotional expressions of different people occurred randomly. The inter-block interval included a fixation cross on a black background, lasting randomly between nine and 11 seconds. To maintain attention, we instructed respondents to press the left or right button according to the gender of the presented face (NgC) or the color of the frame around the picture (NC).

### Functional and structural MRI acquisition and ECG

The acquisition was performed on the Siemens Prisma 3 T MR whole-body scanner with 64-channel head-neck coil. A high-resolution structural T1 image was scanned for each participant. This makes it possible to localize the active brain areas more accurately than using functional images only. Parameters of the MPRAGE sequence were 240 sagittal slices, repetition time (TR) = 2300 ms, echo time (TE) = 2.34 ms, field of view (FOV) = 256 mm, flip angle = 8°, slice thickness = 1 mm). Functional blood-oxygen-level dependent (BOLD) MR data were acquired in a single scanning session with a T2*-weighted multiecho multiband echo-planar imaging (ME MB EPI) sequence of 1250 scans (60 slices, TR = 1000 ms, TE = 14, 34.63 and 55.26 ms, FOV = 200 mm, flip angle = 50°, slice thickness = 2.5 mm, MB factor = 5). TE values were chosen according to recommendations for ME EPI [55], where the second echo was very close to the typical value of single echo acquisition. Simultaneously with the fMRI data, ECG and respiration were measured by Brain Products BrainAmp ExG MR system with 5 kHz sampling frequency. Both signals were corrected for MR gradient artifacts [56] in Brain Vision Analyzer 2 and then used for RETROICOR correction of fMRI data. Processed ECG signal was also used for HRV calculation.

### Data analysis

#### Self-reported data

Self-reported data were analyzed in IBM SPSS Statistic (Version 27) [57]. Independent t-tests were performed to explore group differences in age and in self-report questionnaires. The Mann-Whitney U test was used to assess differences in the level of education between patients and HC.

#### fMRI data pre-processing

All scans were realigned first. The process was applied to middle echo scans when each middle echo scan was aligned to the first middle echo scan. Estimated translations and rotations were used in the realignment procedure of other echoes. RETROICOR technique [58] suppressed any physiological noise originated in ECG and respiration in all scans. Information from all three echoes was used to create a composite scan using the contrast-to-noise weighted average. In each voxel, temporal SNR (tSNR) values were computed for each echo. The resulting voxel value was given by the weighted average of the three original tSNR-weighted values and echoes [59, 60]. Composite functional scans were then coregistered to high resolution structural T1-weighted images and all data were normalized to the Montreal Neurological Institute (MNI) template. As a last step, the spatial smoothing of functional data was calculated by Gaussian filter with full-width at half-maximum (FWHM) of 5 mm.

The data quality was checked for the presence of spatial abnormalities in Mask Explorer [61] and for the presence of excessive movement in the movement_info tool (https://www.nitrc.org/projects/movement_info). The movement in the data was controlled by a framewise displacement (FD) measure [62]. Data from all participants were eligible using the thresholds of 20% of scans exceeding FD = 0.5 mm and 1% of scans exceeding FD = 1.5 mm [59].

#### fMRI data analysis

The data were processed with SPM12 toolbox (http://www.fil.ion.ucl.ac.uk/spm) running under MATLAB R2021a [63]. General linear modelling (GLM) was used to analyze the pre-processed data.

At first, the difference between NgC and NC condition and the comparison between BPD and HC group were examined. GLM was performed on the subject level, where the design matrix contained three time courses of visual stimuli timings convolved with canonical hemodynamic function and six confound regressors for movement (translations and rotations from realignment pre-processing procedure). The task was modelled as a block design, where three time courses represent the NgC blocks, the NC blocks, and the inter-block interval. First-level contrasts correspond to NgC and NC and their difference.

In the next step, group level GLM analysis was performed. We used a two-sample t-test to find the differences between patients with BPD and HC. Group results were evaluated at the cluster level inference with an initial cut-off of *p* < .001 and cluster size larger than 20 and *p* < .05 with FWE correction on the cluster level. For the evaluation of the NgC and NC difference across all measured subjects one-sample t-test was used. The Xjview 10.0 toolbox was used to display results [64].

To assess the effect in the region of interest (ROI) only, the pipeline was the same as for the whole brain analysis. The amygdala region was selected based on masks created from automated anatomical labeling (AAL) atlas. The ROI representative was extracted as a mean of voxels assigned to the ROI. The extraction was performed on contrast estimates (NgC – NC). The medication effect was first shown using the medication index as a covariate in the group level GLM analyses. Then, statistical comparison between contrast estimates with and without medication was computed by paired t-test. For the analysis of amygdala habituation, the task regressors in the subject level GLM model were modulated by linear function, which characterizes the linear change of the effect over time. In the SPM model, the specifications of the first order time modulation were selected for task regressors. Time modulation of regressors allows for the characterization of nonstationary responses, e.g., habituation. We modeled linear effect/linear change of heights in regressors over time. This approach was used, for example, in Peters et al. [65]. The advantage lies in using all scans in one model, which provides a better signal-to-noise input into the model than comparing the average BOLD signal from 18 seconds of first and last blocks. Random order of the blocks (with max 2 repetitions consequently) also didn’t allow to split each subject data into two parts of the same length.

Correlation analyses were conducted to examine associations between amygdala activity, specifically, HRV, and BPD symptoms.

#### Heart rate variability

ECG data were preprocessed in the BrainVision Analyzer 2 [66]. MR gradient artifacts were suppressed by a subtraction approach [56]. The data were resampled to a 250 Hz sampling rate and filtered with the Butterworth zero phase low pass filter with a cut-off at 40 Hz. Then, R waves were detected by a template matching procedure and manually checked with a visual inspection.

The standard deviation of heart rate values was computed in the NgC blocks as the heart rate variability (HRV) parameter. The comparison between the patients with BPD and HC groups were calculated using a Wilcoxon rank sum test in MATLAB R2021a. Correlation analyses were conducted to examine associations between HRV and BPD symptoms.

## Results

The results of the independent t-test showed no significant differences between BPD and HC groups in age. Significant between-group differences were found in BSL-23, A-RSQ, DERS-SF, MDI, and CTQ scores. In all questionnaires, patients with BPD had significantly higher scores than HC. A Mann-Whitney U test suggested a significant difference in education. Details are presented in Table 1. Since there was a significant difference in educational level between groups, the possible effect of education on self-report questionnaires was explored by ANCOVA (using education as a covariate). Results showed no effect of education on differences between the groups in any of the self-report questionnaires.

**Table 1:** Demographic and clinical characteristics of the study sample

	BPD patients		HC		BPD vs HC independent t-test		
	*N* = 30		*N* = 30				
	M	SD	M	SD	T (df)	*p*-value	D
Age	24.2	5.2	24.7	5.2	- 0.3 (58)	0.74	5.2
BSL-23	51.7	14.0	5.7	5.5	16.7 (37.9)	< .001	10.6
A-RSQ	15.3	4.8	6.5	2.8	8.5 (58)	<. 001	4.0
DERS-SF	61.5	7.3	31.0	6.6	16.9 (58)	<. 001	6.9
MDI	72.2	18.0	38.4	7.4	9.5 (38.6)	<. 001	13.7
CTQ	58.1	13.4	32.3	5.8	9.5 (37.8)	<. 001	10.2
Education	N		N		Mann-Whitney U test		
					U	*p*-value	r
Primary	6		4		310	.025	.031
Lower secondary	4		0				
Higher secondary	16		15				
University	4		11				

*BPD* borderline personality disorder, *HC* healthy control, *M* mean, *SD* standard deviation, *BSL-23* Borderline Symptom List, *A-RSQ* Rejection Sensitivity Questionnaire, *DERS-SF* Difficulties in Emotion Regulation Scale Short Form, *MDI* Multiscale Dissociation Inventory, *CTQ* Childhood Trauma Questionnaire

### fMRI results

The between-group comparison did not show any significant whole-brain differences between patients with BPD and HC in the NgC contrasted to the NC. Further, we performed a region of interest (ROI) analysis focused on the differences between groups specifically in the amygdala. The ROI analysis revealed no significant differences between patients and controls in amygdala activation.

**Fig. 1:**
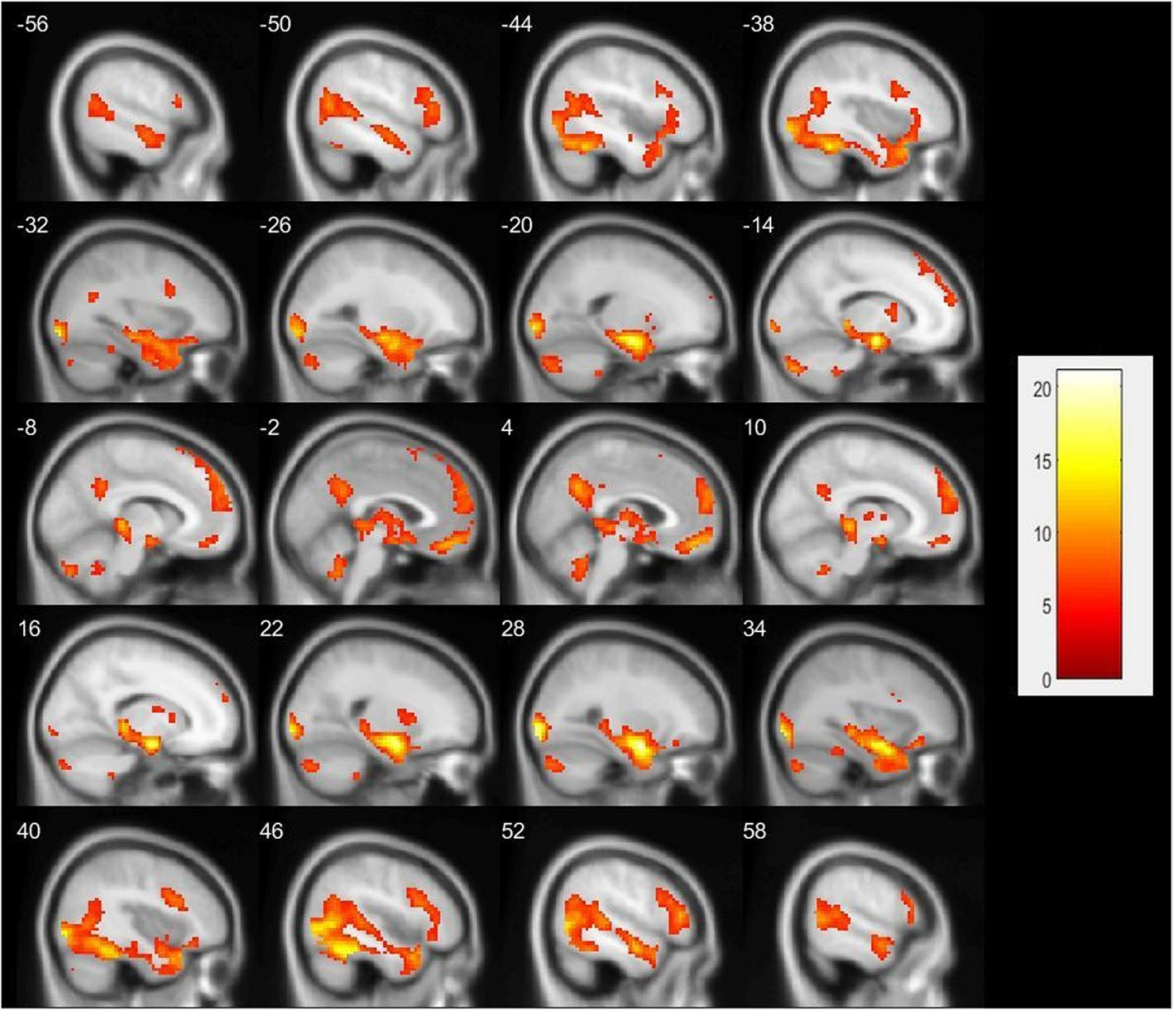
Activation map showing areas with higher activation during negative facial processing in all subjects (patients with BPD and HC together). Shown areas represent higher activity at threshold p(FWE) < .05 (contrast NgC minus NC)

As we identified no differences in fMRI between the groups, we compared the task conditions on the whole sample. The cluster-level whole-brain analysis identified eight large clusters in the NgC contrasted to NC condition (contrast NgC minus NC). Clusters had activation peaks in the cerebellum posterior lobe, hippocampus, thalamus, amygdala, medial frontal gyrus, putamen, superior and frontal gyrus, precuneus, and cingulate gyrus (Fig. 1). A detailed description of clusters is provided in Table 2.

**Table 2:** fMRI whole-brain results associated with negative facial expression processing as compared to scrambled pictures (contrast NgC minus NC) in all respondents. Results significant on the cluster level with FWE correction *p* < .05

Anatomic label	Side L/R	Peak MNI coordinate			Peak intensity (t)	Cluster size	P_FWE-cor_
		X	Y	Z			
Cerebellum posterior lobe	L	−3	−58	−44	8.92	168	<.001
Cerebellum posterior lobe	L	−15	− 82	−41	10.57	158	<.001
Hippocampus, thalamus, amygdala	R	27	−1	−20	21.07	6156	<.001
Cerebellum posterior lobe	R	24	−82	−35	8.30	119	<.001
Medial frontal gyrus	Medial	0	47	−17	12.39	234	<.001
Putamen	L	−12	11	4	7.44	44	<.001
Superior frontal gyrus, medial frontal gyrus	R	9	59	28	9.67	552	<.001
Precuneus, cingulate gyrus	R	3	−58	34	10.71	268	<.001

The anatomic label of the peak voxel is shown. *BPD* borderline personality disorder, *HC* healthy control, *L* left, *R* right, *MNI* Montreal Neurological Institute (x, y and z coordinates are provided in mm), *Cluster size* the number of voxels, *NgC* negative condition (negative emotional faces). *NC* neutral condition (scrambled pictures)

To assess the effect of educational level, statistical comparison between contrast estimates (NgC minus NC) with and without educational level as a covariate was computed by paired t-test. Results showed no significant differences.

### Additional analyses

#### Medication effect

**Fig. 2:**
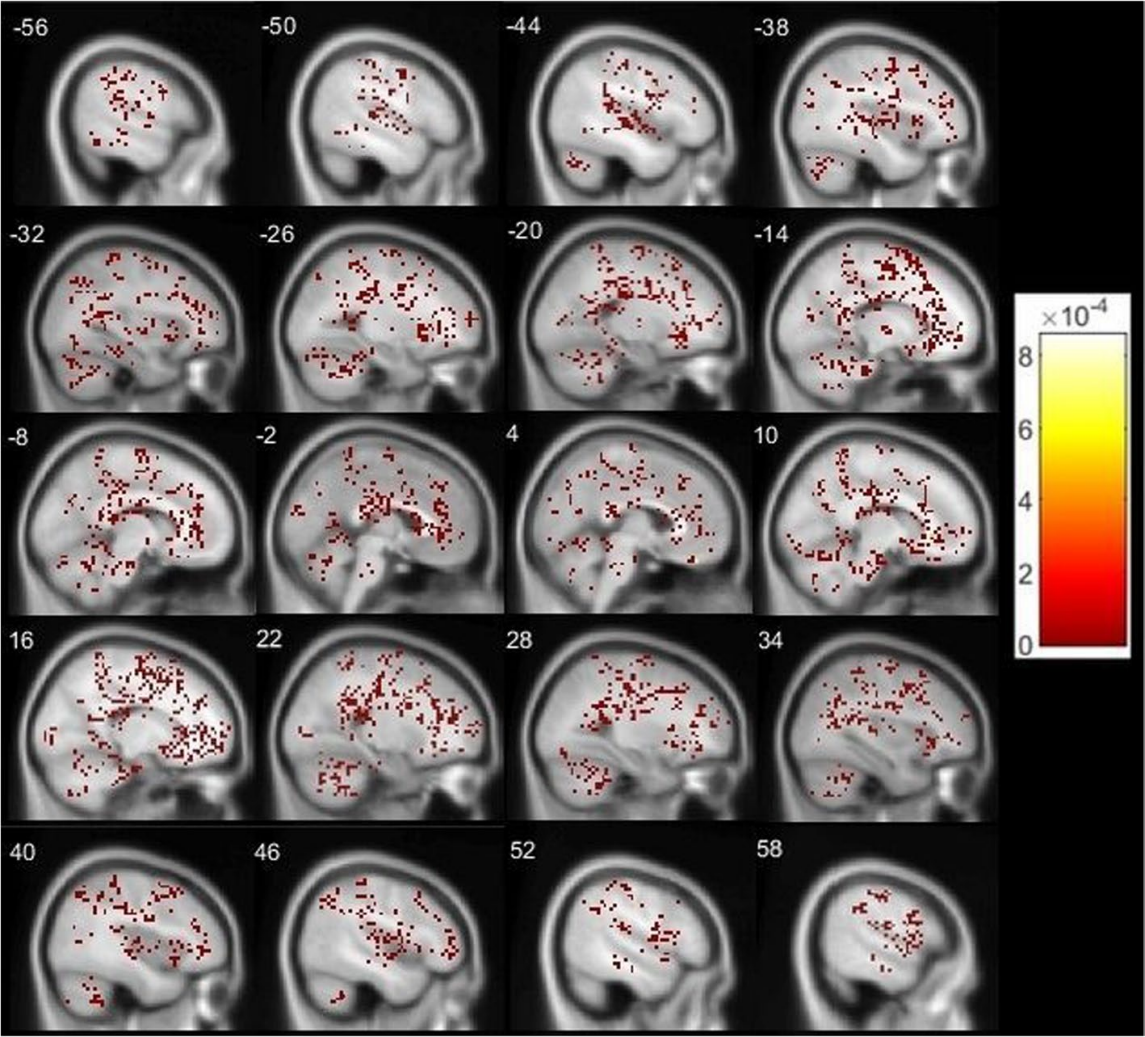
Results of the comparison of contrast estimates (NgC minus NC) with and without medication

The results showed no significant differences between contrast estimates (NgC minus NC) with and without medication (Fig. 2).

#### Amygdala habituation

Some authors have suggested that it might not be that the average amygdala activity is impaired in patients with BPD during emotional stimuli, but rather that patients with BPD show lower habituation on emotional stimuli as compared with HC [67, 68]. Since we did not find differences in amygdala activity between the groups, we also explored differences in amygdala habituation. The group comparison results did not show differences in amygdala habituation between the patients with BPD and HC (t = 4.55; p_FWE_ = .47).

### Heart rate variability results

**Fig. 3:**
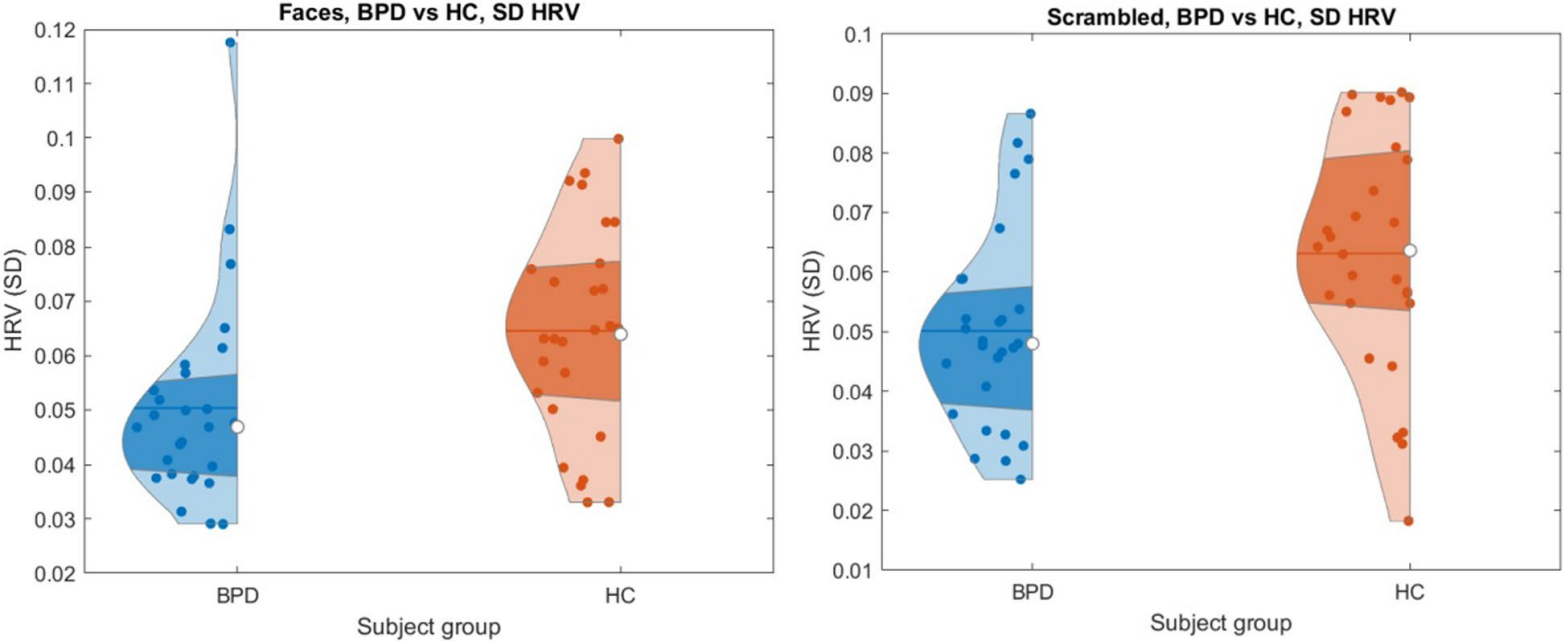
Differences between patients with BPD and HC during the NgC (*p* = .004; *r* = .35) and NC (*p* = .003; *r* = .35) condition

The results showed significantly lower HRV in patients with BPD than in HC (*p* = .004; *r* = .35) in NgC condition, as illustrated in Fig. 3. HRV was significantly lower in patients also in NC condition (*p* = .003, *r* = .35). No differences were found within-groups in NgC versus NC condition (BPD group: *p* = .70; *r* = .007; HC group: *p* = .52; *r* = .03).

### Correlation analyses

#### Amygdala activity and HRV

No significant correlations were found between the left amygdala and HRV (*r* = .12, *p* = .53) and the right amygdala and HRV (*r* = .11, *p* = .56) in BPD patients. No significant correlations were found between the left amygdala and HRV (*r* = −.01, *p* = .95) and the right amygdala and HRV (*r* = .09, *p* = .65) in healthy controls.

#### Amygdala activity and self-report questionnaires

Since no differences were found in amygdala activity between groups, correlations were performed on the whole sample. No significant correlations were found between the left amygdala activity and BSL-23 (*r* = −.08; *p* = .54), A-RSQ (*r* = −.07; *p* = .58), MDI (*r* = −.07; *p* = .55), DERS-SF (*r* = − .03; *p* = .79), or CTQ (*r* = − .06; *p* = .61) scores. No significant correlations were found between the right amygdala activity and BSL-23 (*r* = −.20; *p* = .11), A-RSQ (*r* = − .02; *p* = .87), MDI (*r* = −.13; *p* = .31), or DERS-SF (*r* = −.13; *p* = .32) scores.

#### Heart rate variability and self-report questionnaires

No significant correlations were found between HRV and BSL-23 (*r* = −.15; *p* = .43), A-RSQ (*r* = .15; *p* = .43), MDI (*r* = −.19; *p* = .31), DERS-SF (*r* = .33; *p* = .09), and CTQ (*r* = .04; *p* = .81) scores in patients with BPD. As well in HC, no significant correlations were found between HRV and BSL-23 (*r* = −.00; *p* = .99), A-RSQ (*r* = .08; *p* = .67), MDI (*r* = .24; *p* = .22), DERS-SF (*r* = −.04; *p* = .83), or CTQ (*r* = .17; *p* = .38).

## Discussion

The current study explored fMRI and HRV correlates of negative emotional facial processing in patients with BPD and HC. We found large scale activation across the brain in the whole sample while processing negative facial expressions as compared to the control condition (scrambled pictures). Contrary to our hypotheses, we found no significant differences between patients with BPD and HC in neural activity nor higher amygdala activity specifically in patients with BPD as compared with HC when processing negative facial expressions as compared to the control condition. Moreover, amygdala activity was unrelated to BPD symptom severity. The results suggest no abnormal BOLD activity during negative emotional facial processing in patients with BPD. Further, patients with BPD displayed lower HRV as compared with HC, regardless of condition type. However, there was no significant association between HRV and BPD symptoms, rejection to sensitivity, dissociation, emotional dysregulation, and childhood trauma as measured by self-report questionnaires both in patients with BPD and HC. These findings indicate that low HRV may be associated with BPD, but it was unrelated to experimental condition and symptoms assessed in this study.

In the whole sample, we found heightened activity in the cerebellum posterior lobe, hippocampus, thalamus, amygdala, medial frontal gyrus, putamen, superior frontal gyrus, precuneus, and cingulate gyrus. Activity in the superior frontal gyrus, precuneus, hippocampus, amygdala, putamen, and medial frontal gyrus was also found in previous studies exploring brain activity during facial processing in HC and patients with BPD [12, 15, 21, 22]. Thus, the “faces” task seems a valid method for eliciting the activity of areas connected to facial emotion processing. However, differences between patients with BPD and HC were not detected by the “faces” task in this study, although the differences in self-reported BPD symptoms between the groups were very high.

Several factors may explain the discrepancy between our findings and previous findings regarding brain activity and mainly amygdala activity during facial processing in patients with BPD. Previous studies found amygdala hyperactivity in patients while viewing neutral and fearful expressions [5, 12, 17]. In the current study, we presented respondents with mixed expressions of fear, disgust, and sadness. Wrege et al. [17] pointed out that the neural substrates of emotion processing seem to be task-dependent and affected by the type of comparison. Thus, it is possible that combining negative expressions of different emotions might have diminished amygdala hyperactivity or brain differences between groups in general. Moreover, there seem to be fMRI correlates during facial expression experiencing other than the amygdala. For example, studies found heightened activity of the superior frontal gyrus [17, 18, 21], medial frontal gyrus [12, 15], hippocampus [18, 69], and precuneus [12, 17, 18]. Other studies did not find heightened amygdala activity [18, 20]. It is possible that amygdala activity is heightened primarily in processing neutral or fearful expressions; therefore, various emotional valences should be explored separately. Moreover, altered brain activity to neutral faces in BPD is a stable finding [5, 17]; using neutral faces as a control condition for comparing to negative faces might be therefore biased. It follows that the relationship between higher amygdala activity and processing negative facial expressions is not straightforward.

According to some studies [67, 68], differences between patients with BPD and HC in emotion processing are not connected to amygdala hyperactivity but rather to slower amygdala habituation in patients. Following this hypothesis, we also analyzed differences in amygdala habituation to investigate the possibility of this effect. However, we did not find significant differences in habituation, thus we can rule out this process in our study and support previous ideas.

Studies by Frick et al. [70] and Mier et al. [71] that identified amygdala hyperactivity in patients with BPD during neutral and negative expressions used tasks in which the respondents were instructed to identify the presented facial emotion. Following the procedure from Paret et al. [37], respondents in our task were instructed to identify gender on the facial pictures to maintain their attention while viewing the faces. It is possible that neural correlates identified in previous studies might have reflected the emotion recognition process, not only the emotional experience while viewing faces. However, further research is needed to explore how ways of maintaining attention may affect these tasks. We may speculate that passive viewing of emotional expression is insufficient to detect abnormalities in brain processing of emotional faces in patients with BPD, even though Donegan et al. [19] found heightened amygdala activity even in this type of task. It is also possible that identifying gender on the face pictures might have diminished the power to detect differences between the groups.

Another possible influence in our study is patient medication. According to Schulze, Schmahl and Niedtfeld [72], medication considerably influences neural activations of the left amygdala and hippocampus. In their study, medication-free patients displayed hyperresponsivity to negative stimuli compared to HC. However, no hyperresponsivity was found in patients with BPD currently taking various psychotropic medication. This suggestion was supported by a later study by Paret et al. [37], in which a single dose of citalopram reduced bilateral amygdala activity while viewing negative affective expressions in the same task that was used in the current study. Since we included mostly medicated patients (83.3%) in our study, we analyzed the possible effect of medication. However, we did not find significant differences in neural activity with and without medication. Thus, we can rule out the effect of the medication on neural activity in this study. Nevertheless, we cannot rule out the effect of the medication completely since we had one group with various kinds of medications and the second group without any medication.

This study’s second focus was examining HRV differences during facial emotion processing. Compared to HC, patients exhibited lower HRV when viewing both negative expressions and scrambled pictures. This finding suggests that patients generally have a lower HRV compared to HC. However, we did not find a significant relationship between HRV and amygdala activity in the patient group. The absence of this relationship might be explained by no differences in amygdala activity between patients and healthy controls. Also, there was no relationship between HRV and BPD symptom severity, rejection sensitivity, dissociation, emotional dysregulation, or childhood trauma. This result contrasts with previous studies [33–35, 73, 74]. A possible explanation may lie in methodological differences. The studies that found a positive relationship between HRV and BPD measured HRV outside the fMRI, whereas we measured HRV in fMRI. Although we did not clarify the association between lower HRV and BPD symptoms, low HRV seems to be connected to a worsened ability to react to emotional stimuli [33–35] and it might underlie disturbed emotion processing in BPD. A possible explanation for why group differences were found in HRV and not in fMRI may lie in the RETROICOR preprocessing technique. This routinely used technique suppresses the variability associated with physiological noise in the context of fMRI data, including the heart pulsation. On the basis of the timing of R-waves in the ECG, basic functions are regressed from fMRI, which suppresses undesired physiological artifacts and enhances the signal-to-noise ratio [58, 75]. In our case, a partial suppression of the desired effect might occur. In general, it seems appropriate to assess emotional dysregulation by various markers, including both fMRI and psychophysiological functions. According to our expectations, BPD patients have lower HRV in general. Although we still do not know what processes underlie lower HRV in patients, it was not task dependent in our study.

Several limitations of this study should be discussed. As mentioned, we used the “faces” task with a combination of negative expressions instead of focusing on one specific emotion. Presenting more than one emotion and quickly changing among several expressions may affect neural activity and diminish potential differences. Although this may be understood as a limitation, this “faces” task design is diverse and complex, thus resembling social interactions in everyday life.

Because of our sample composition, we cannot generalize results to men. We also did not exclude patients with BPD with comorbid disorders. Therefore, we cannot reject the possibility that the absence of diversity in neural activity and lower HRV are associated with patient comorbidities. However, comorbid disorders are natural in patients with BPD. Moreover, most of our BPD patients were medicated, thus we cannot rule out the medication influence completely, and further studies could include a direct comparison of medicated and unmedicated patients.

Previous studies and reviews [5, 12, 17] provided evidence about abnormal amygdala activity of patients with BPD during facial emotion processing. However, we did not find any differences in brain activity. The relationship between BPD symptoms and the amygdala seems more complex, and more research is needed. Future research should explore neural activity connected to specific emotional valence rather than multiple emotions. It is also necessary to clarify the role of medication in changing neural activity and comorbidity in HRV abnormality. Although low HRV seems connected to emotional instability and worsened ability to react to emotional stimuli [33–35], more studies are needed to better understand its relationship.

## Conclusions

To our knowledge, this is the first study exploring brain activity together with heart rate variability and their relationship during facial emotion processing in BPD patients. We did not find any differences between patients and healthy controls in brain activity, neither in amygdala, specifically. Further, there were no difference in amygdala habituation during the task between the groups. Although heightened amygdala activity is a frequently reported result, it seems that its connection to facial emotion processing is not straightforward, and future studies should explore its activity during specific emotional valence rather than multiple emotions. The results showed lower HRV in patients with BPD than in healthy controls, but HRV was not associated with BPD symptoms. Therefore, low HRV seems to be connected to the emotion dysregulation disorder, however its relationship to specific symptoms or functions needs to be clarified.

## Data Availability

The datasets used and analyzed during the current study are available from the corresponding author upon reasonable request.

## Abbreviations

AAT: Approach Avoidance Task
ACC: Anterior cingulate cortex
A-RSQ: Rejection Sensitivity Questionnaire
BOLD: Blood-oxygen-level dependent
BPD: Borderline personality disorder
BSL-23: Borderline Symptom List
CAN: Central autonomic network
CTQ: Childhood Trauma Questionnaire
DERS-SF: Difficulties in Emotion Regulation Scale Short Form
DMN: Default mode network
DSM-V: Diagnostic and Statistical Manual of Mental Disorders, Fifth Edition
ECG: Electrocardiogram
FD: Framewise displacement
FDR: False discovery rate
fMRI: Functional magnetic resonance imaging
FOV: Field-of-view
FWE: Family-wise error
FWHM: Full width at half maximum
GLM: General linear modelling
HC: Healthy control
HRV: Heart rate variability
L: Left
M: Mean
MDD: Major depressive disorder
MDI: Multiscale Dissociation Inventory
ME MB EPI: Multiecho multiband echo-planar imaging
MNI: Montreal Neurological Institute
N: Number of participants
NC: Neutral condition
NgC: Negative condition
OCD: Obsessive-compulsive disorder
PFC: Prefrontal cortex
PTSD: Posttraumatic stress disorder
R: Right
ROI: Region of interest
SCID-5-PD: Structured Clinical Interview for DSM-5 Personality Disorders
SD: Standard deviation
TE: Time to echo
TR: Repetition time
vmHRV: Vagally mediated heart rate variability
